# Variable Tandem Glycine-Rich Repeats Contribute to Cell Death-Inducing Activity of a Glycosylphosphatidylinositol-Anchored Cell Wall Protein That Is Associated with the Pathogenicity of Sclerotinia sclerotiorum

**DOI:** 10.1128/spectrum.00986-23

**Published:** 2023-05-04

**Authors:** Yawen Hu, Hang Gong, Ziyang Lu, Pengpeng Zhang, Sinian Zheng, Jing Wang, Binnian Tian, Anfei Fang, Yuheng Yang, Chaowei Bi, Jiasen Cheng, Yang Yu

**Affiliations:** a College of Plant Protection, Southwest University, Chongqing City, China; b Key Laboratory of Agricultural Biosafety and Green Production of Upper Yangtze River, Ministry of Education, Southwest University, Chongqing City, China; c State Key Laboratory of Agricultural Microbiology, Huazhong Agricultural University, Wuhan City, China; Zhejiang University - Zhijiang Campus

**Keywords:** *Sclerotinia sclerotiorum*, pathogenicity, cell wall, cell death-inducing activity, tandem repeats, allelic variants

## Abstract

Glycosylphosphatidylinositol (GPI) anchoring of proteins is a conserved posttranslational modification in eukaryotes. GPI-anchored proteins are widely distributed in fungal plant pathogens, but the specific roles of the GPI-anchored proteins in the pathogenicity of Sclerotinia sclerotiorum, a devastating necrotrophic plant pathogen with a worldwide distribution, remain largely unknown. This research addresses *SsGSR1*, which encodes an *S. sclerotiorum* glycine- and serine-rich protein named SsGsr1 with an N-terminal secretory signal and a C-terminal GPI-anchor signal. SsGsr1 is located at the cell wall of hyphae, and deletion of *SsGSR1* leads to abnormal cell wall architecture and impaired cell wall integrity of hyphae. The transcription levels of *SsGSR1* were maximal in the initial stage of infection, and *SsGSR1*-deletion strains showed impaired virulence in multiple hosts, indicating that *SsGSR1* is critical for the pathogenicity. Interestingly, SsGsr1 targeted the apoplast of host plants to induce cell death that relies on the glycine-rich 11-amino-acid repeats arranged in tandem. The homologs of SsGsr1 in *Sclerotinia*, *Botrytis*, and *Monilinia* species contain fewer repeat units and have lost their cell death activity. Moreover, allelic variants of *SsGSR1* exist in field isolates of *S. sclerotiorum* from rapeseed, and one of the variants lacking one repeat unit results in a protein that exhibits loss of function relative to the cell death-inducing activity and the virulence of *S. sclerotiorum*. Taken together, our results demonstrate that a variation in tandem repeats provides the functional diversity of GPI-anchored cell wall protein that, in *S. sclerotiorum* and other necrotrophic pathogens, allows successful colonization of the host plants.

**IMPORTANCE** Sclerotinia sclerotiorum is an economically important necrotrophic plant pathogen and mainly applies cell wall-degrading enzymes and oxalic acid to kill plant cells before colonization. In this research, we characterized a glycosylphosphatidylinositol (GPI)-anchored cell wall protein named SsGsr1, which is critical for the cell wall architecture and the pathogenicity of *S. sclerotiorum*. Additionally, SsGsr1 induces rapid cell death of host plants that is dependent on glycine-rich tandem repeats. Interestingly, the number of repeat units varies among homologs and alleles of SsGsr1, and such a variation creates alterations in the cell death-inducing activity and the role in pathogenicity. This work advances our understanding of the variation of tandem repeats in accelerating the evolution of a GPI-anchored cell wall protein associated with the pathogenicity of necrotrophic fungal pathogens and prepares the way toward a fuller understanding of the interaction between *S. sclerotiorum* and host plants.

## INTRODUCTION

Glycosylphosphatidylinositol (GPI) anchoring of proteins is a conserved posttranslational modification in eukaryotes, including protozoa, fungi, plants, insects, and mammals (1). Upon GPI modification, the GPI attachment signal peptide at the carboxyl termini consisting of ethanolamine phosphate, trimannoside, glucosamine, and inositol phospholipid is replaced by a preassembled GPI in the endoplasmic reticulum (2). GPI-anchored proteins (GPI-APs) were then transported from the endoplasmic reticulum to the plasma membrane. In fungi, a large subset of GPI-APs are then transferred from the fungal membrane to the cell wall. These are known as GPI-anchored cell wall proteins (GPI-CWPs), while a small number of GPI-APs remain located at the plasma membrane of fungi and are known as GPI-anchored plasma membrane proteins (GPI-PMPs) (3–5).

As a dynamic structure that protects the cells from changes in various environmental stresses, fungal cell walls are mainly comprised of chitin, β-1,3-glucan, and glycoproteins (6). In many ascomycetes, GPI-APs form the largest group of cell wall proteins and act as adhesins, enzymes, or sensors of cell wall integrity (3, 4). GPI-APs are also widely distributed in fungal plant pathogens and are found to accumulate in appressoria and invasive hyphae (7). However, the roles of GPI-APs in plant-pathogenic fungi have only recently been reported. The Aspergillus flavus GPI-anchored protein encoded by *ecm33* participates in growth, development, aflatoxin biosynthesis, and maize infection (8). Targeted deletion of the Botrytis cinerea *BcCFEM1* gene, which encodes a CFEM domain-containing protein with a GPI-anchored site, resulted in impaired virulence, conidial production, and stress tolerance (9). Recently, it was reported that a Ser/Thr-rich GPI-anchored protein, SGP1, is required for the pathogenicity of Ustilaginoidea virens during panicle infection and that SGP1 functions as a recognizable pathogen-associated molecular pattern (PAMP) and triggers innate immunity in rice leaves (10).

Tandem repeated sequences, also known as minisatellite or microsatellite DNA, can trigger frequent recombination events and lead to expansion and contraction of gene size due to polymorphisms of repeat units and, in turn, alter the amino acid sequences of the corresponding protein (11). Variable repeats confer higher rates of evolution of genes and their phenotypes, including cell surface variability, plasticity in skeletal morphology, and tuning of the circadian rhythm (12). Interestingly, many cell surface proteins in fungi have been reported to contain tandemly arranged repeats. The variability in the number of tandem repeats in four GPI-modified adhesins was frequently described in clinical Candida albicans isolates, indicating that the repeats may allow cells to adapt to a fluctuating environment and thus play a role during infection (13, 14). The speculation was partially confirmed by comparative analysis of the cellular binding specificity of C. albicans isolates containing adhesin *ALS3* alleles with 12 tandem repeat copies or 9 copies (15). A genome analysis of Saccharomyces cerevisiae revealed that several cell wall proteins contain tandem repeats with size variation, which creates quantitative alteration in phenotypes, such as adhesion, flocculation, and biofilm formation (16). Nevertheless, the specific roles of tandem repeats in the majority of GPI-CWPs during host plant-fungal pathogen interactions remain largely unknown.

Sclerotinia sclerotiorum is a devastating fungal pathogen with a worldwide distribution. This pathogen infects more than 400 plant species and threatens several important crops, such as oilseed rape, sunflower, soybean, and some vegetables (17). *S. sclerotiorum* was believed to be a typical necrotrophic pathogen for a long time, and the fungus mainly applies cell wall-degrading enzymes and oxalic acid to kill host cells (18). Recent evidence indicates that the successful infection by *S. sclerotiorum* involves a potential biotrophic growth at the initial stage (19). Since the release of genome information and the development of genetic transformation technology for *S. sclerotiorum*, several genes have been functionally characterized in recent years. These genes mainly encode proteins that are related to the oxidative stress response (20, 21), act as effectors that suppress host immunity (22, 23), or degrade chemical compounds of resistance (24, 25), suggesting a complex and subtle interaction between *S. sclerotiorum* and its host plants.

Cell walls of *S. sclerotiorum* have long been examined and characterized. The hyphal walls of the fungus consist of β-glucan, chitin, and some glycoproteins, which are similar to those found in the cell walls of other fungi (26). A trifluoromethanesulfonic acid (TFMS) analysis of the *S. sclerotiorum* cell wall proteome revealed 24 GPI-CWPs and 30 non-GPI-CWPs, and some of them were encoded only in the *Sclerotinia* and *Botrytis* genomes (27). At the same time, only a few of the cell wall proteins of *S. sclerotiorum* have been functionally characterized. *S. sclerotiorum* cell wall protein Ss-Sl2 contains two PAN modules and is essential for sclerotial development and cellular integrity (28). Some secreted proteins may localize to the cell wall of *S. sclerotiorum*, including SSITL, which is known to act as a potential effector and is critical for pathogenicity (29). Considering that GPI attachment signal peptides have yet to be described in Ss-Sl2 and SSITL, the roles of GPI-CWPs in *S. sclerotiorum* remain unknown.

In this study, a gene named *SsGSR1* in *S. sclerotiorum* (*SS1G_11413*) is predicted to encode a GPI-CWP rich in glycine and serine residues. Our findings show that *SsGSR1* is not only required for the cell wall architecture of hyphae but also critical for the pathogenicity of *S. sclerotiorum*. Moreover, the encoded protein SsGsr1 works as a cell death-inducing protein (CDIP) relying on the glycine-rich tandemly arranged repeats. Interestingly, the exact number of repeat units showed variation between individuals and species, creating alterations in cell death-inducing activity and the role in pathogenicity. These results can advance our understanding of the variation and function of GPI-CWPs with tandem repeats in necrotrophic fungal plant pathogens.

## RESULTS

### *SsGSR1* is critical for the virulence of *S. sclerotiorum*.

**FIG 1:**
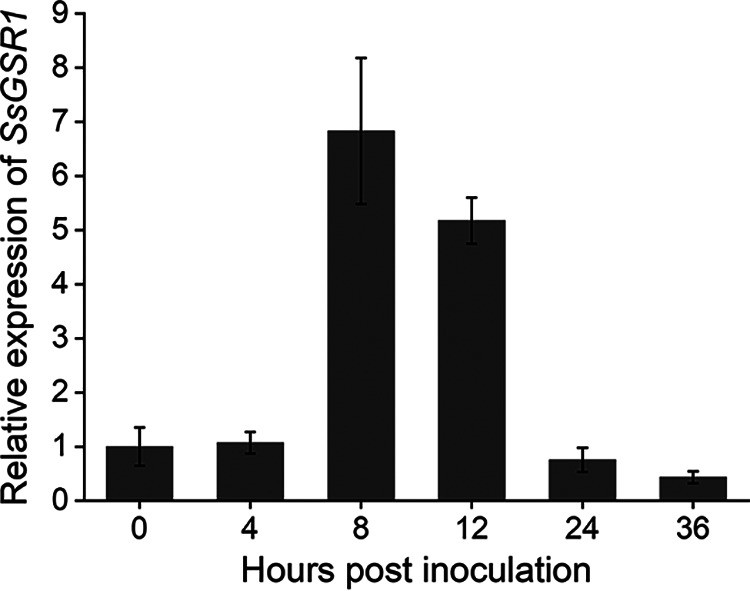
The expression pattern of *SsGSR1* during infection. The relative expression level of *SsGSR1* is upregulated after contact with the leaves of N. benthamiana. Total *SsGSR1* cDNA abundance in the samples was normalized using the β-tubulin gene as a control. The relative expression of *SsGSR1* in mycelium inoculated on plants at 0 h was set as 1. Three independent replicates were performed. Error bars represent the standard deviation of the mean.

**FIG 2:**
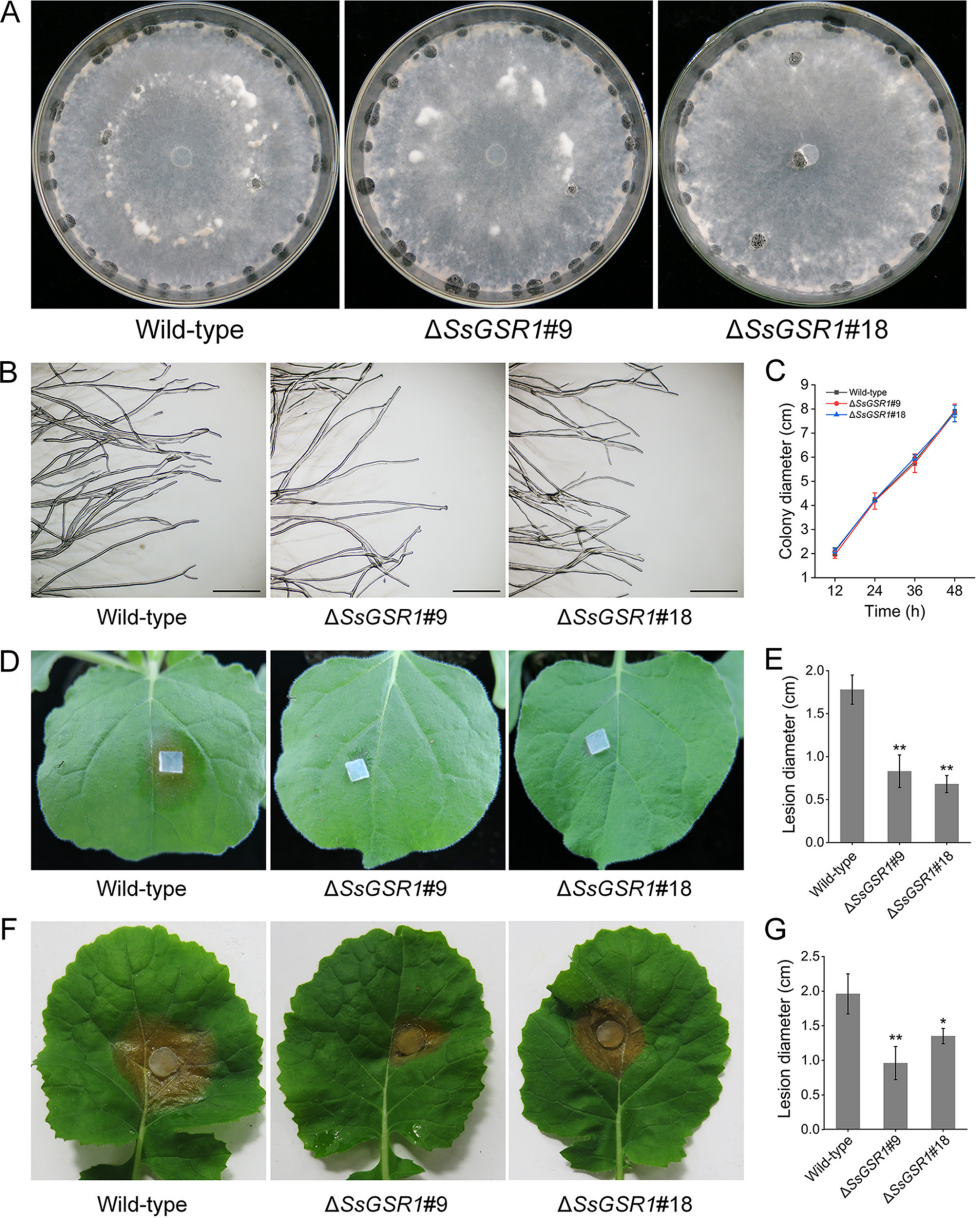
*SsGSR1* is critical for the virulence of *S. sclerotiorum*. (A) Colony morphology of *SsGSR1* gene-deletion strains. (B) Branching pattern of the *SsGSR1* gene-deletion strains grown on PDA medium. Bar, 0.5 mm. (C) Radical growth of the *SsGSR1* deletion strains. The strains were cultured on PDA, and the colony diameters were measured at 12-h intervals. Each point represents a single experiment’s mean and standard deviation from four independent cultures. The experiment was repeated twice with similar results. (D to G) Virulence assays of the *SsGSR1* gene-deletion strains. (D and E) Photographs and average lesion diameters of the gene-deletion strains on leaves of N. benthamiana at 24 h postinoculation (hpi). (F and G) Photographs and average lesion diameters of the *SsGSR1* deletion strains on leaves of *B. napus* at 48 hpi. Error bars represent standard deviations. Statistical significance was analyzed using a Student *t* test between the wild type and each gene-deletion strain (*, *P* < 0.05; **, *P* < 0.01).

For functional analysis of *SsGSR1* in the pathogenicity of *S. sclerotiorum*, the expression levels of *SsGSR1* during infection by *S. sclerotiorum* were evaluated. The transcription levels of *SsGSR1* were maximal in the initial stage of infection of Nicotiana benthamiana, suggesting that the gene is related to the pathogenicity of *S. sclerotiorum* (Fig. 1). The gene was then disrupted using a split-marker strategy. Two gene-deletion mutants, Δ*SsGSR1*#9 and Δ*SsGSR1*#18, were obtained and verified by PCR and reverse transcription-PCR (RT-PCR) analysis (see Fig. S1 in the supplemental material). When cultured on potato dextrose agar (PDA) medium for 15 days, the colony morphology of the *SsGSR1* gene-deletion strains was similar to that of the wild-type strain (Fig. 2A). The branching pattern and the growth rate of the hyphae for the *SsGSR1* gene-deletion mutants were identical to those of the wild-type strain (Fig. 2B and C). To determine whether deletion of *SsGSR1* would confer a change in pathogenic capability of *S. sclerotiorum*, the gene-deletion strains were inoculated on leaves of tobacco (Nicotiana benthamiana) and oilseed rape (Brassica napus). The results showed that the two mutants exhibited reduced virulence on the leaves of tobacco (Fig. 2D and E) and oilseed rape (Fig. 2F and G). These evidences indicated that *SsGSR1* is critical for the virulence of *S. sclerotiorum*.

### SsGsr1 is located at the cell wall of the hyphae.

**FIG 3:**
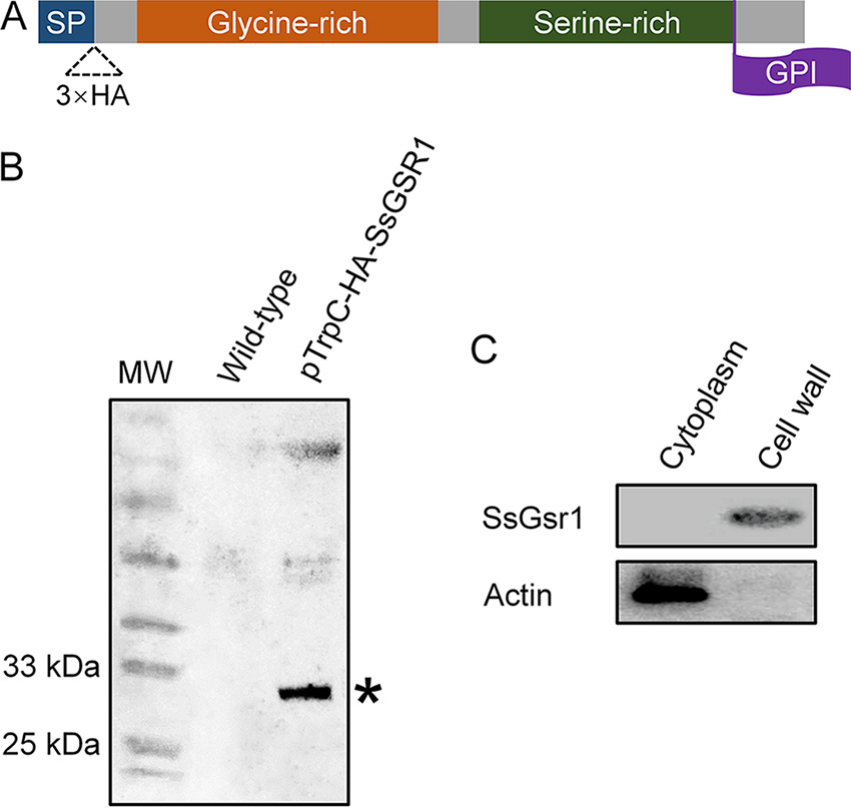
SsGsr1 is located at the cell wall of *S. sclerotiorum*. (A) Diagram of the construct for the localization analysis of SsGsr1. The secretory signal peptide (SP) and the glycine-rich and serine-rich regions are represented in blue, red, and green, respectively. The site of the GPI anchor addition (the ω site) was marked in purple. The 3×HA fragment was inserted after the SP of SsGsr1. (B) Generation of the strain encoding HA-tagged SsGsr1. The total proteins of the strain PtrpC-HA-SsGSR1 and the wild-type strain were extracted separately and subjected to Western blotting with anti-HA antibodies. MW, molecular weight. Asterisk indicates HA-tagged SsGsr1. (C) SsGsr1 was detected in the cell wall of hyphae. The cell wall protein and the cytoplasm protein in the mycelium of PTrpC-HA-SsGSR1 were extracted separately and subjected to Western blot analysis using anti-HA (top) or anti-actin (bottom) antibodies.

The 18 initial N-terminal amino acids of SsGsr1 were predicted to make up a signal peptide with the SignalP-5.0 Server (http://www.cbs.dtu.dk/services/signalp/). The protein contains a C-terminal GPI-anchor signal sequence, and the omega site position is amino acid S^231^ when predicted with PredGPI (http://gpcr.biocomp.unibo.it/predgpi/) (Fig. 3A). The protein also contains a glycine-rich region at the N-terminal region (G^33^ to G^133^) and a serine-rich region at the C-terminal region (S^147^ to S^232^), which are often presented in GPI-CWPs of yeast fungi (30, 31). Thus, we inferred that the SsGsr1 protein is anchored to the outer layer of the plasma membrane and located at the cell wall. To confirm this, we generated a hemagglutinin (HA)-tagged SsGsr1-engineered *S. sclerotiorum* strain named PTrpC-HA-SsGSR1. The total proteins of the strain were subjected to Western blot analysis with an anti-HA antibody. The results showed that a protein migrated at approximately 30 kDa, consistent with a predicted mass for the HA-SsGsr1 protein of 27 kDa (Fig. 3B). For the next step, the strain’s cytoplasmic and cell wall proteins were extracted, respectively, and subjected to Western blot analysis. SsGsr1 could be detected in the cell wall but not in the cytoplasm (Fig. 3C), indicating that SsGsr1 is located at the cell wall of the hyphae of *S. sclerotiorum*.

### *SsGSR1* is involved in the cell wall architecture and integrity.

**FIG 4:**
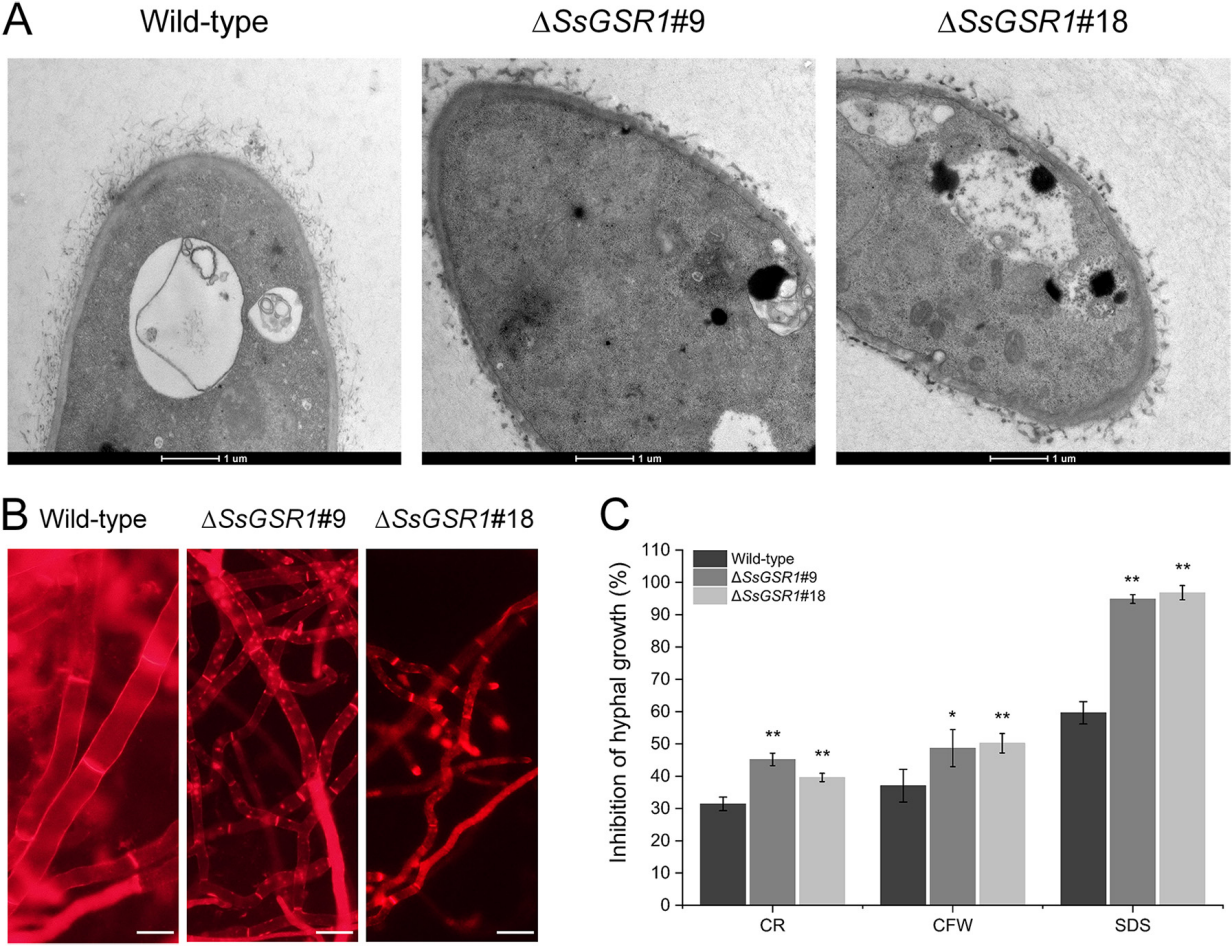
*SsGSR1* is related to the cell wall architecture and integrity of *S. sclerotiorum*. (A) Transmission electron microscopy of *SsGSR1* gene-deletion strains. (B) Microscopy of Congo red-stained material (red dots) along the hyphae of the *SsGSR1*-deletion strain. Bar, 10 μm. (C) Sensitivity of the *SsGSR1*-deletion strains to cell integrity perturbation agents. The deletion strains were inoculated on PDA plates amended with 0.4 g/L Congo red (CR), 0.2 g/L calcofluor white (CFW), and 0.015% sodium dodecyl sulfate (SDS). The inhibition of hyphal growth was calculated at 36 hpi. Bars indicate standard deviations. Statistical significance was analyzed using a Student *t* test between the wild type and each gene-deletion strain (*, *P* < 0.05; **, *P* < 0.01).

Since SsGsr1 is localized in the cell wall of *S. sclerotiorum*, we wonder whether it might be involved in the cell wall architecture. The cell wall morphology of the hyphae for the two gene-deletion strains was observed by transmission electron microscopy. The hyphae of the two mutants exhibited noncontinuous dark deposits at the outer wall (Fig. 4A). The hyphae of the *SsGSR1* gene-deletion strain were stained with Congo red (CR), a dye with a high affinity for β-1,3-glucan (32). Strongly stained cell wall patches were detected along the hyphae of the *SsGSR1*-deletion strains. At the same time, the hyphae of the wild-type strain were homogeneously labeled (Fig. 4B). These results indicate an abnormal deposition of the cell wall in the hyphae due to the deletion of *SsGSR1*.

A further experiment was carried out to compare the cell wall integrity between the wild-type and *SsGSR1* deletion strains. The strains were cultured on PDA amended with different cell wall perturbation agents, and then the hyphal growths were evaluated. Two gene-deletion strains appeared to be more sensitive to CR, calcofluor white (CFW), and sodium dodecyl sulfate (SDS) (Fig. 4C), indicating an impaired cell wall integrity due to the loss of *SsGSR1*. In conclusion, *SsGSR1* is involved in the architecture of the cell wall and the integrity of *S. sclerotiorum.*

### SsGsr1 targets the apoplast of N. benthamiana to induce cell death and trigger PAMP-triggered immunity (PTI) responses.

**FIG 5:**
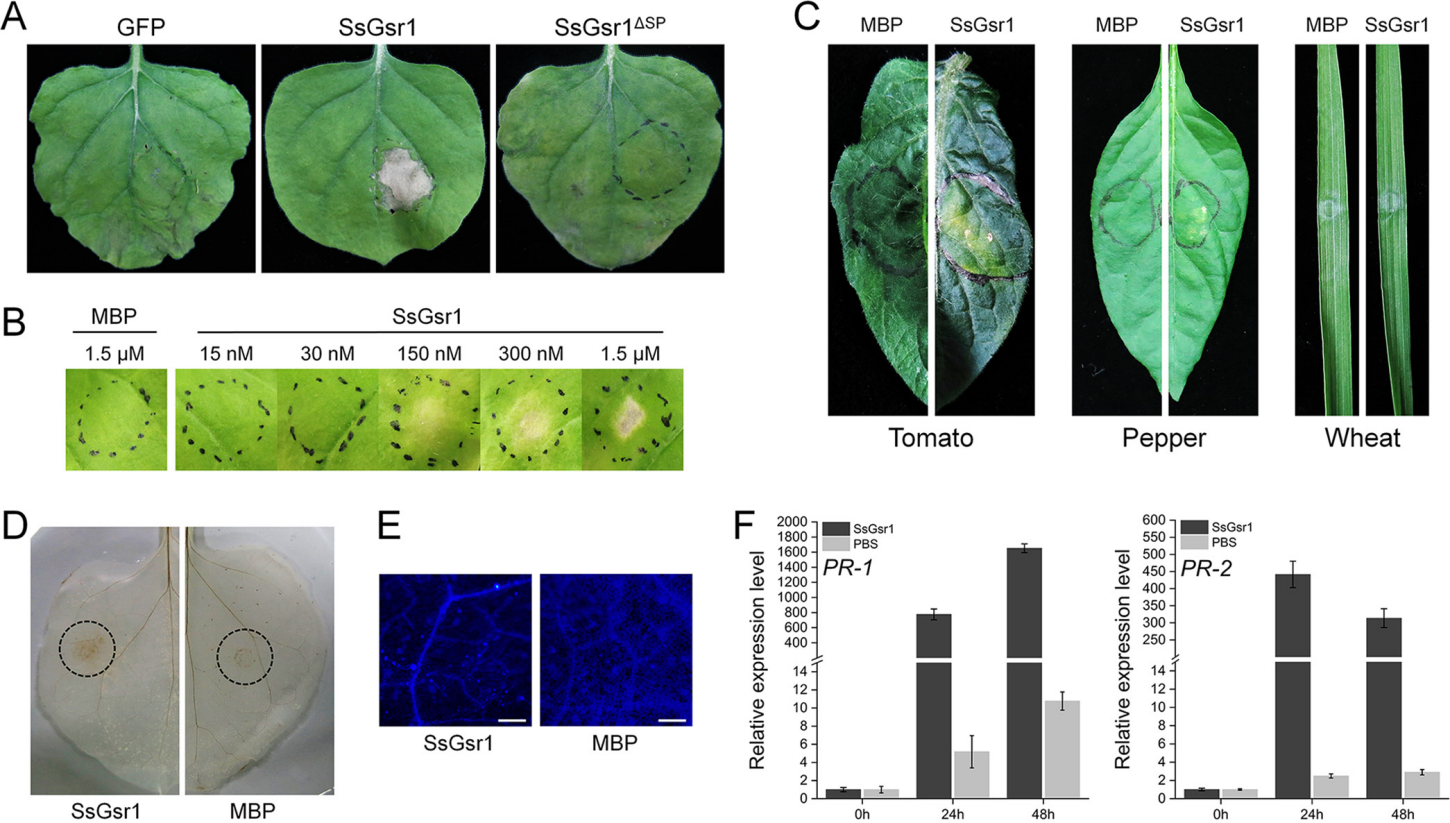
SsGsr1 triggers cell death and PTI responses. (A to C) SsGsr1 targeted the apoplast of N. benthamiana to induce cell death. (A) Representative N. benthamiana leaves 5 days after agroinfiltration using constructs containing SsGsr1 or SsGsr1 without secretory signal peptide (SsGsr1^ΔSP^). The green fluorescent protein (GFP) was agroinfiltrated as a control. (B) Representative N. benthamiana leaves infiltrated with purified SsGsr1 (15 nM to 1.5 μM). Purified maltose-binding fusion protein (MBP; 1.5 μM) was infiltrated as a control. (C) Treatment of tomato, pepper, and wheat with 1.5 μM purified SsGsr1 proteins. The images were taken 2 days postinfiltration for panel B and 5 days postinfiltration for panels A and C. (D to F) SsGsr1 triggers PTI responses. (D and E) ROS accumulation (D) and callose formation (E) in N. benthamiana that was infiltrated with the SsGsr1 protein solution (1.5 μM). The MBP (1.5 μM) was infiltrated and used as a control. Bar, 200 μm. (F) The expression level of *PR-1* and *PR-2* in the leaves of N. benthamiana 0, 2,4 and 48 h after infiltration with the SsGsr1 protein solution (1.5 μM) or phosphate-buffered saline (PBS). The N. benthamiana actin gene in each tissue was used as an internal control. The relative expression levels of *PR-1* or *PR-2* at 0 h were set as 1. Three independent replicates were performed. Error bars represent the standard deviations.

Recent research indicates that some fungal GPI-CWPs could induce cell death in plants (10). To test whether SsGsr1 has similar activity, the protein was transiently expressed in N. benthamiana using *Agrobacterium*-infiltration method analysis. SsGsr1 induced cell death in leaves of N. benthamiana, while the control vector did not (Fig. 5A). To test whether SsGsr1 targets the apoplast to trigger cell death, the N-terminal secretory signal peptide was removed to produce SsGsr1^Δsp^. Transient protein expression in N. benthamiana demonstrated that SsGsr1^Δsp^ did not have cell death-inducing activity (Fig. 5A). Then, *SsGSR1* was expressed in Escherichia coli using the pMAL-c2X vector. The recombinant SsGsr1 protein was tested for cell death-inducing activity by infiltrating 30 nM to 1.5 μM protein solution into the leaves of N. benthamiana. The results showed that the protein could induce rapid cell death of N. benthamiana, the degree of which was increased with increasing concentrations of SsGsr1 from 150 nM to 1.5 μM (Fig. 5B). The cell death-inducing activity of the recombinant SsGsr1 protein was also observed with additional dicot plants, including tomato and pepper, but not monocot plants, such as wheat (Fig. 5C).

Recent research has reported that some apoplast cell death-inducing proteins are recognized by the plant immune system, triggering PAMP-triggered immunity (PTI) (33, 34). Infiltration of N. benthamiana with purified SsGsr1 protein solutions resulted in rapid accumulation of reactive oxygen species (ROS) and callose deposition (Fig. 5D and E). Two pathogenesis-related (PR) genes, *PR-1* and *PR-2*, were also dramatically expressed in response to the SsGsr1 protein (Fig. 5F). Taken together, these results indicated that SsGsr1 targets the apoplast of N. benthamiana to induce cell death and can activate PTI responses.

### Tandem repeats in SsGsr1 are responsible for inducing cell death.

**FIG 6:**
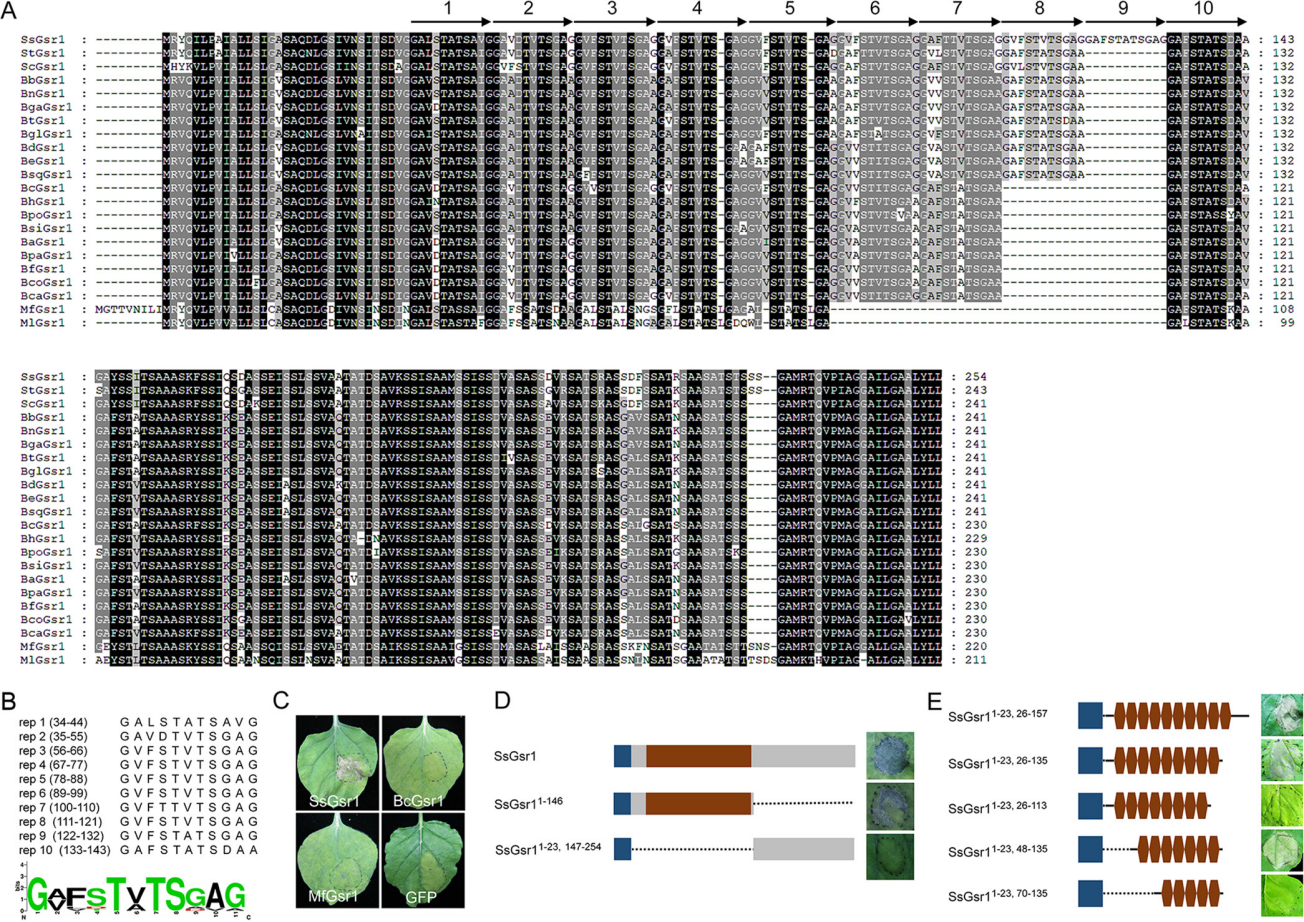
Tandemly arranged 11-amino-acid repeats in SsGsr1 are responsible for inducing cell death. (A) Multialignment of the amino acid sequences of SsGsr1 homologs from species of *Sclerotinia*, *Botrytis*, and *Monilinia*. Shading indicates sequence similarities of 100% (dark), 75% (medium), and 50% (light). Arrowheads indicate the tandem arranged repeat units. (B) Sequence alignment of the 10 tandem repeat units of SsGsr1. The amino acid frequency plots are shown at the bottom. (C) Representative N. benthamiana leaves 5 days after agroinfiltration using constructs encoding *B. cinerea* BcGsr1 and *M. fructicola* MfGsr1. (D) Representative N. benthamiana leaves 5 days after agroinfiltration using constructs that contain various truncated SsGsr1 proteins. Blue, secreted signal peptide; red, region containing tandem repeats. (E) Representative N. benthamiana leaves 5 days after agroinfiltration using constructs that contain various number units of the tandem repeat of SsGsr1. Blue, secreted signal peptide; red, tandem repeat units. The dotted lines in panels D and E indicate gaps in the constructs.

The homologs of SsGsr1 were distributed mainly in Ascomycetes, especially the species in Helotiales (Fig. S2). Amino acids of SsGsr1 homologs from species of *Sclerotinia*, *Botrytis*, and *Monilinia* were selected to construct multiple alignments (Table S1). SsGsr1 exhibited high similarity to homologs of species of *Sclerotinia*, *Botrytis*, and *Monilinia* in the N- and C-terminal regions (Fig. 6A). However, the homologs showed greater variability in the regions rich in glycine. For these homologs, different numbers of amino acids are missing in these regions compared with SsGsr1. The number of missing amino acids is 11 for Sclerotinia trifoliorum StGsr1, 11 or 22 for homologs of *Botrytis* species, and 44 for homologs of two *Monilinia* species. All of these numbers are multiples of 11. A close examination revealed that the lost fragment is part of a tandemly arranged repeat of a unit sequence of 11 amino acids (reps 1 to 10, shown by the arrows in Fig. 6A). The alignment of the tandem repeats in SsGsr1 and the amino acid frequency plots (WebLogos) are shown in Fig. 6B. The repetitive sequences are identical in seven units (reps 3 to 10); substitution for only one amino acid could be found in rep 7 and rep 9. However, reps 1, 2, and 10 showed more variation in amino acid level. The number of tandem repeats varied between the SsGsr1 homologs. SsGsr1 contains one to two repeats more than that of the homologs in *Botrytis* species and four repeats more than that in two *Monilinia* species. For the next step, *B. cinerea* BcGsr1 and Monilinia fructicola MfGsr1 were transiently expressed in N. benthamiana through agroinfiltration. Both the homologs could not induce cell death of N. benthamiana (Fig. 6C).

Given the difference in the number of tandem repeats among the SsGsr1 homologs, we hypothesized that the region containing the tandem repeats is responsible for the cell death-inducing activity of SsGsr1. Thus, we assayed the two truncated mutants, testing their ability to trigger cell death by agroinfiltration in N. benthamiana. The results confirmed that a truncated protein containing the tandem repeats could induce rapid cell death (Fig. 6D). For the next step, the different truncations of the tandem repeat-containing fragment were linked with the secretory signal peptide directly and used for agroinfiltration of the leaves of N. benthamiana. The amino acids 48 to 135, which contain reps 3 to 9, were necessary for cell death-inducing activity (Fig. 6E). Taken together, these results indicated that the tandem 11-amino-acid repeats are critical for the cell death activity of SsGsr1.

### Tandem repeats of *SsGSR1* in field insolates of *S. sclerotiorum* vary in size and generate functional variability.

**FIG 7:**
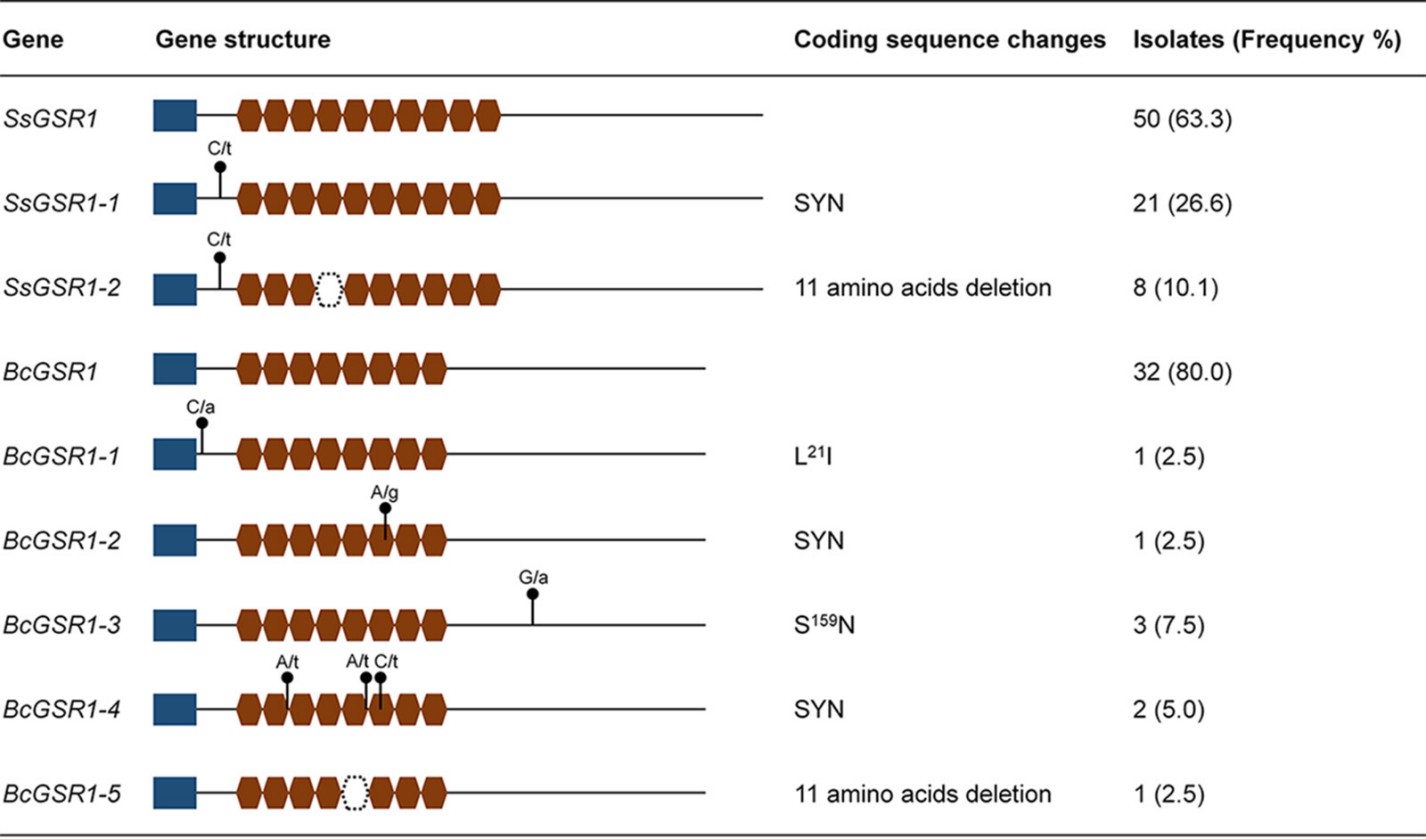
Allelic variants of *SsGSR1* and *BcGSR1* exist in the natural population. Single nucleotide polymorphisms are indicated as N/n, where N represents the nucleotide in strain 1980 and n represents the replacement nucleotide. The dotted lines indicate gaps in the alignments. SYN, synonymous amino acid substitutions. The blue box represents the secretory signal peptide. The red box represents the 33-bp unit that corresponds to the 11-amino-acid repeats.

Because of the great diversity of selective pressures on cell interactions, genes that encode the cell wall proteins of fungi evolve fast and show rapid divergence (35). Thus, the allelic variation in *SsGSR1* in the field population of *S. sclerotiorum* was analyzed. The DNA sequence for *SsGSR1* was sequenced for 79 isolates collected in rapeseed stem rot in nine districts of Chongqing City, China, as described by Yu et al. (36). Interestingly, two types of DNA modifications, labeled *SsGSR1-1* and *SsGSR1-2*, were identified in the open reading frames (ORFs) of *SsGSR1*. In *SsGSR1-1*, a synonymous mutation (84C>T) in DNA sequences was observed with high frequency in the collections (26.6%). In *SsGSR1-2*, in addition to the synonymous mutation, a 33-bp fragment that exactly corresponded to rep 4 was deleted in the nucleotide sequence of *SsGSR1* in eight isolates (10.1%) (Fig. 7). We also tested the *BcGSR1* polymorphisms in the natural population of *B. cinerea*. These results indicated that frequency modifications were observed in the coding region of *BcGSR1* of a small and random selection of field strains isolated in Chongqing City. It is interesting that a 33-bp fragment that corresponds to one repeat unit was also deleted in one isolate.

**FIG 8:**
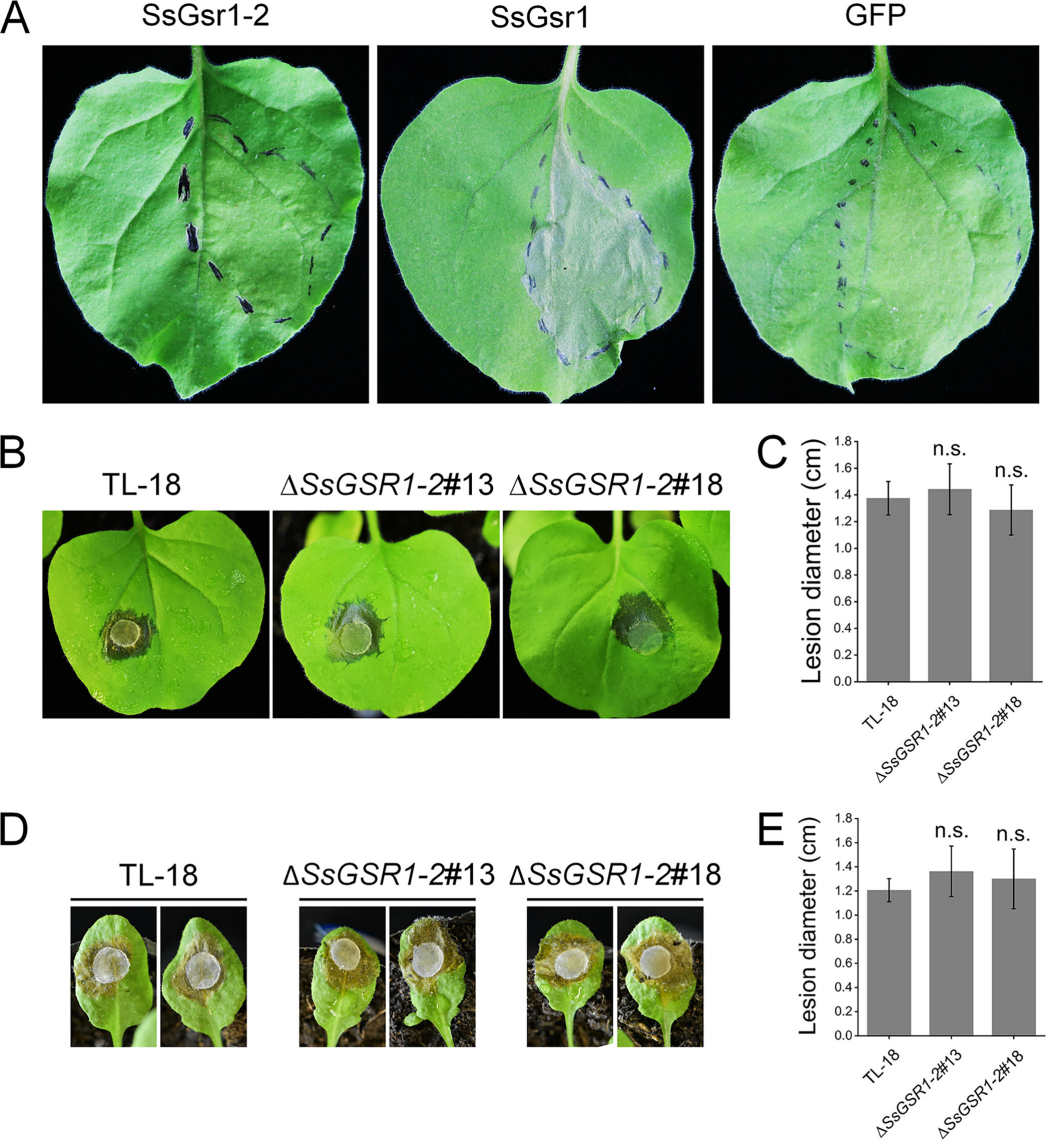
*SsGSR1-2* allele exhibited loss of function relative to cell death-inducing activity and the pathogenicity of *S. sclerotiorum*. (A) Representative N. benthamiana leaves 5 days after agroinfiltration using constructs encoding SsGsr1-2. SsGsr1 and GFP were used as positive and negative controls, respectively. (B to E) Virulence assay of the *SsGSR1-2* gene-deletion strains. (B and C) Photographs and average lesion diameters of the gene-deletion strains on leaves of N. benthamiana at 12 hpi. (D and E) Photographs and average lesion diameters of the gene-deletion strains on leaves of *A. thaliana* at 24 hpi. Bars indicate standard deviations. “n.s.” means there is no significant difference.

To test whether the amino acid modifications affect the cell death-inducing activity, the allele *SsGSR1-2* was transiently expressed in N. benthamiana by agroinfiltration. The encoded protein SsGsr1-2 could not induce cell death (Fig. 8A), which is consistent with the finding that the fragment containing reps 3 to 9 is critical for cell death-inducing activity. These results led us to question the role of the allele *SsGSR1-2* in the pathogenicity of *S. sclerotiorum*. To perform a functional analysis of the role of the allele of *SsGSR1-2*, the gene was deleted from an isolate named TL-18 that was separated from oilseed rape in the Tongliang district of Chongqing City. Two gene-deletion strains were obtained and confirmed with PCR and RT-PCR analysis (Fig. S3). The strains were then inoculated on leaves of N. benthamiana and Arabidopsis thaliana. The results showed that the two mutants displayed virulence similar to that of the wild-type strain (Fig. 8B to E). These results indicate that the allelic variants of *SsGSR1* exist in the natural population and that one of the variants results in the protein that exhibited loss of function relative to cell death-inducing activity and the virulence of *S. sclerotiorum*.

## DISCUSSION

Fungal cell walls are dynamic structures and organelles that are critical for cell viability, morphogenesis, and pathogenesis. GPI-APs have long been reported to locate at the cell wall of fungi, especially yeast species, and involve cell-cell interactions such as flocculation, biofilm formation, mating, or adhesion to the host cell surface (3, 4). However, the roles of GPI-APs were shown to be essential for the pathogenicity of fungal plant pathogens only recently (9, 10). In this research, *S. sclerotiorum* SsGSR1 is predicted to contain an N-terminal secretory signal and a C-terminal GPI-anchoring signal and is located on the cell wall of hyphae, suggesting that it belongs to GPI-CWPs. The protein was also predicted to contain glycine- and serine-rich regions. When considering the complete protein sequence, four types of amino acids account for more than 10% of the total amino acids, including serine (23.23%), alanine (18.5%), glycine (13.78%), and threonine (11.81%). The subcellular location of SsGsr1 is consistent with previous reports that GPI-APs containing long regions rich in serine and threonine residues exhibit a cell wall location (37). The deletion of *SsGSR1* led to altered virulence of *S. sclerotiorum* in different host plants, indicating that *SsGSR1* is critical for the pathogenicity of *S. sclerotiorum*. This evidence suggests an association between GPI-CWPs and the pathogenesis of filamentous fungal pathogens.

Most cell walls of fungi are layered. The inner cell wall consists of a core of covalently attached branched β-1,3-glucan with 3% to 4% interchain and chitin, while the outer layers vary much more than the inner layer and are rich in mannoproteins for Ascomycetes (3). GPI-CWPs are usually covalently bound to β-1,3-glucans through β-1,6-glucans and located at the outer layer, while previous evidence indicated that they are not essential in establishing a three-dimensional (3D) polysaccharide cell wall network (38). In this research, deletion of *SsGSR1* of *S. sclerotiorum* led to a mutation in the outer layer of the cell wall of the hyphae since the hyphae of the *SsGSR1* gene-deletion strains exhibited noncontinuous dark deposits in the outer layer of the cell wall. Such mutation has not affected the hyphal growth under normal conditions but impairs the ability to maintain the integrity of the cell wall, since the hyphal growth of the *SsGSR1* deletion strains was shown to be more sensitive to different cell wall perturbation agents than that of the wild-type strain.

The molecular mechanism of SsGsr1 involved in the development of the cell wall is still unclear. The strongly stained patches were detected along the hyphal cell wall of the *SsGSR1* gene-deletion strains when stained with Congo red, which binds to β-linked-glucans or chitins (39). Reactivity to Congo red staining of the cell wall in *SsGSR1* gene-deletion strains suggests the presence of amorphous polysaccharides. Similar Congo red-stained patches on the cell wall of hyphae were also reported in Phytophthora parasitica *CBEL* gene-silencing strains. *CBEL* encodes a cell wall protein with two cellulose binding domains and is involved in cell wall deposition (40, 41). In this research, SsGsr1 contains a glycine-rich region in the N-terminal region. Interestingly, glycine-rich proteins in plant cell walls have been proposed to act as scaffolds or agglutinating agents for the deposition of cell wall constituents (42). SsGsr1 is hypothesized to be involved in the structural organization of core polysaccharides in *S. sclerotiorum*, and the mutations may also affect the outer layer formation of the cell wall, such as the retention of cell wall mannoproteins.

The transient expression of SsGsr1 in leaves of N. benthamiana led to cell death, which was confirmed by infiltrating the recombinant SsGsr1 protein. Thus, SsGsr1 was considered to be one of the CDIPs. Following a hypothesized biotrophic stage, *S. sclerotiorum* has a dominant necrotrophic phase during infection (19). Traditionally, the pathogen mainly relies on cell wall-degrading enzymes (CWDEs) and oxalic acid to kill its hosts and feeds on dead or dying tissue for nutrition. Recent evidence indicates that necrotrophic pathogens release highly redundant plant CDIPs to infect many host plants (43). To date, some CDIPs have been reported for *S. sclerotiorum*, including SsCP1 (23), SsNep1 and SsNep2 (44), and SsNE1 to SsNE6 (45), and most of these CDIPs are secretory proteins. As we know, SsGsr1 is the first GPI-CWP in necrotrophic fungal pathogens that acts as a CDIP. GPI-CWPs are usually covalently bound to β-glucans, the main constituents of the cell wall of hyphae of *S. sclerotiorum* (26). During plant colonization, the first fungus-derived structure that physically contacts the host cell is the cell wall of the hyphae. Host plants secret a large plethora of hydrolytic enzymes, including chitinases and glucanases, into the apoplast upon fungal attack, and these lead to the degradation of the hyphal cell wall. It is possible that SsGsr1 could be released from the cell wall during the infection processes and then induce cell death, which is critical for the infection by *S. sclerotiorum*.

SsGsr1 contains tandemly arranged repeats in the glycine-rich region, and the fragment containing rep units 3 to 9 is a response to the cell death-inducing activity. The reps 3 to 9 represent the core repeat unit since they are identical at the amino acid levels. Accordingly, the natural allele *SsGSR1-2* that has lost the DNA fragment corresponding to rep 4 cannot induce cell death. Tandem repeats were also conserved in the SsGsr1 homologs, which are mainly distributed in Helotiales of Ascomycetes, especially in *Botrytis* and *Monilinia* species. However, the number of repeats showed variation among these homologs. Compared with SsGsr1, BcGsr1 and MfGsr1 lost reps 8 and 9 and reps 6 to 9, respectively. BcGsr1 and MfGsr1 could not induce cell death in plants, further suggesting that the core tandem repeats are responsible for cell death-inducing activity. In addition to the tandem repeats, SsGsr1 also contains a serine-rich region at the C-terminal region. The C-terminal region of Candida glabrata GPI-CWP adhesin Epa1p is rich in serine and threonine, and this region helps the N-terminal domain to project away from the body of the cell and function in the extracellular environment (31). Since the C-terminal region does not participate in the cell death activity of SsGsr1 during agroinfiltration in N. benthamiana, it may facilitate the function of cell death of the tandem repeats during the infection processes.

Genes that encode the cell wall proteins of fungi evolve fast and show rapid divergence, due to the diversity of selective pressures on cell interaction (36). Previous research has shown that several genes that encode the cell wall in Saccharomyces cerevisiae contain tandem repeat sequences, and some of them are highly polymorphic in length in the wild population (16, 46). It is highly possible that the gene length polymorphism is due to the different molecular mechanisms (particularly replication slippage or the repair of double-stranded breaks during DNA replication) proposed for minisatellite array expansion and contraction (47, 48). In this research, we report the identification of an allele, *SsGSR1-2*, with one fewer tandem repeat unit than *SsGSR1*. The isolates containing the *SsGSR1-2* allele were widely distributed in two genetically distinct subpopulations in Chongqing City (37). Similar polymorphisms were also detected in *BcGSR1* of some field strains of *B. cinerea* isolated from Chongqing, which may indicate that the acquisition and loss of minisatellites have been fast during evolution. The minisatellite sequence that has a variable number of tandem repeats is also responsible for the polymorphism of homologs in species of *Sclerotiorum*, *Botrytis*, and *Monilinia*. Interestingly, the lower the degree of relatedness to *S. sclerotiorum*, the fewer the repeat units, suggesting that the tandem repeats are related to the evolution of the species in Sclerotiniaceae, allowing adaptation to the environment.

Although minisatellites have long been used for phylogenetic tying of eukaryotes, the elements have historically been designated nonfunctional “junk” DNA. In recent years, minisatellite sequences have been found often to occur in adhesin or flocculation genes in both pathogenic and nonpathogenic yeast species (49, 50). This research provides direct evidence linking the minisatellites and virulence-related genes of fungal plant pathogens. Considering that SsGsr1 plays an important role in cell wall development and infection processes, an interesting question would be whether the generation and maintenance of different *SsGSR1* alleles offer *S. sclerotiorum* isolates an adaptive advantage in response to the environmental stress and the host plant defense response. In Saccharomyces cerevisiae, the size variation in minisatellites in genes that encode cell wall proteins creates quantitative alterations in phenotypes, such as adhesion, flocculation, or biofilm formation (16). An observed change in ORFs of yeast cell wall protein-encoding gene *SED1* affects the number of potential N-glycosylation sites and cysteine residues and thus affects the cell wall properties (51). In this investigation, it is not yet known whether changes in ORFs affect the properties of the cell wall. However, the allele SsGsr1-2 lost the cell death-inducing activity, and deletion of *SsGSR1-2* in field isolates leads to no effect on the virulence of *S. sclerotiorum*. Fungal and oomycete pathogens may have evolved strategies to avoid host recognition by altering or losing their PAMPs and microbial associated molecular patterns (MAMPs) (52). It is hypothesized that the selection of the *SsGSR1-2* allele is an escape from host recognition, since the product of this gene acts as a PAMP. However, such an alteration leads to the loss of the cell death-inducing activity of SsGsr1-2, which is an important approach to the successful infection of necrotrophic pathogens.

## MATERIALS AND METHODS

### Fungal strains and culture conditions.

The Sclerotinia sclerotiorum strain 1980 was used as the wild-type strain unless otherwise stated. The fungal strains used in this study were cultured on potato dextrose agar (PDA) at 20°C and stored at 4°C for long-term preservation. All the transformants were maintained on PDA supplemented with hygromycin B at 100 μg/mL.

### Nucleic acid extraction and manipulation.

To collect fungal hyphae, the mycelia were grown on PDA overlaid with cellophane membranes at 20°C. DNA extraction was based on the cetyltrimethylammonium bromide method as described previously (53). DNA concentrations were determined spectrophotometrically, and extracted DNA was stored at −20°C. Total RNA was extracted with TRIzol reagent (Tiangen, Beijing, China) following the manufacturer’s protocol and treated with DNase I (TaKaRa, Dalian, China) to remove DNA contamination. First-strand cDNA was synthesized following the instructions of the RevertAid first-strand cDNA synthesis kit (Thermo Fisher Scientific, Waltham, MA).

To evaluate gene expression levels, relative quantification was performed with SYBR green RT-PCR on a qTOWER2.0 real-time system (Analytik Jena AG, Germany). The relative expression levels of the *S. sclerotiorum* β-tubulin gene and the N. benthamiana actin gene were used to normalize the RNA sample. The primers were designed across or flanking an intron (see Table S2 in the supplemental material). The cycling conditions were as follows: initial denaturation at 95°C for 2 min, followed by 40 cycles at 95°C for 20 s, primer annealing at 57°C for 15 s, and primer extension at 72°C for 20 s. Four replicates were used. The relative quantitative method (threshold cycle [2^−ΔΔ^*^CT^*]) was used to evaluate the quantitative variation. For each gene, the assays were repeated at least twice, each repetition with three independent replicates.

### Generation and characterization of *S. sclerotiorum* transformants.

*SsGSR1* and the allele *SsGSR1-2* were deleted from the genome of *S. sclerotiorum* through a spit-marker strategy. Briefly, the 5′ untranslated region (UTR) and the 3′ UTR fragments of the *SsGSR1* gene were amplified using the *S. sclerotiorum* genomic DNA as the template. The two fragments were inserted into pSKH (54) in the SacІ/SalІ and KpnІ/SmaІ sites, respectively, and the vector was named pSKH-SsGSR1. Then, the vector pSKH-SsGSR1 was used as a template to amplify two split-marker fragments that overlap the hygromycin phosphotransferase (*HPH*) gene. These two fragments were used to transform the protoplasts of the wild-type strain 1980 or TL-18 by the polyethylene glycol (PEG) method (55). The transformants were selected in regeneration agar medium with 100 mg/mL hygromycin B and purified through a hyphal-tip transfer at least three times. The gene-deletion strain was verified by amplifying the genome DNA with the primer pair designed beyond the 5′ untranslated region and the 3′ UTR fragments of *SsGSR1*. All primers are shown in Table S2.

To evaluate the cell wall integrity of *SsGSR1* gene-deletion strains, the transformants and the wild-type strain were cultured on PDA medium with 0.4 g/L Congo red (CR), 0.2 g/L calcofluor white (CFW), and 0.015% sodium dodecyl sulfate (SDS). The colony diameters were then measured at 36 h to calculate the inhibition of hyphal growth. Each experiment was repeated at least three times.

Pathogenicity assays of the transformants were performed on *A. thaliana*, N. benthamiana, and *B. napus* Qingyou 1. The plants were cultured in a growth chamber at 25°C with a 12-h day/night cycle. Mycelial plugs were cut from the edge of wild-type strains or gene-deletion strains and inoculated on the leaves of the plants. The inoculation trial of each strain was repeated at least five times. The inoculated plants were kept in a greenhouse with 90% relative humidity.

### Protein localization analysis.

To determine the protein localization of SsGsr1, an HA-tagged SsGsr1-engineered *S. sclerotiorum* strain was obtained. First, the gene with a coding sequence of a 3×HA tag that was inserted after the secretory signal peptide was artificially synthesized (Sango, Shanghai, China). The fragment was amplified and inserted into pSilent-1 (56) at the KpnІ/HindШ cloning sites. The resulting HA-tagged *SsGSR1* overexpression vector was linearized with SpeІ and used to transform into the wild-type strain of *S. sclerotiorum* using the PEG method. The cytoplasm and cell wall proteins of the transformant were extracted according to the method of Pitarch et al. (57). Protein samples (50 μg) were separated on an SDS-polyacrylamide gel and transferred to a polyvinylidene difluoride (PVDF) membrane (Millipore, MA, USA) using a semidry transblot electrophoretic transfer system (Bio-Rad, USA). The membrane was blocked in 5% (wt/vol) nonfat powdered milk dissolved in TBS-T (0.1% Tween 20, 20 mM Tris, 150 mM NaCl, pH 7.5) overnight at 4°C. The membranes were then inoculated with anti-HA antibodies or antiactin antibodies (diluted 1:1,000 in TBS-T) for 2 h on a shaker. After washing three times, the membranes were incubated with secondary antibodies (diluted 1:5,000 in TBS-T) for 60 min. Signals were detected using the ECL system (ECL Western blotting substrate; Sango, Shanghai, China) and photographed using the ChemiDoc MP system (Bio-Rad, USA).

### PVX plasmid construction and *Agrobacterium*-mediated transient expression assay.

The *SsGSR1* gene, the *SsGSR1* gene without signal peptide, and truncated *SsGSR1* were amplified from the cDNA of the wild-type strain 1980 of *S. sclerotiorum*. *SsGSR1-2* was amplified from the cDNA of the *S. sclerotiorum* strain TL-18. *BcGSR1* and *MfGSR1* were amplified from the cDNA of *B. cinerea* and *M. fructicola*, respectively. The primers are shown in Table S2. All of these fragments were inserted into pGR106. The recombinant vector was transformed into Agrobacterium tumefaciens strain GV3101 (pSOUP). Single colonies were picked and cultured in liquid LB medium containing the corresponding resistance at 28°C with shaking at 250 rpm. A. tumefaciens cells were collected by centrifugation at 4,000 rpm for 15 min and resuspended in infiltration buffer until the optical density at 600 nm (OD_600_) was around 0.6. The suspensions were incubated for 2 to 3 h at room temperature and infiltrated into the lower epidermis of N. benthamiana leaves. Each assay was performed on six leaves and repeated at least two times.

### Expression and purification of recombinant SsGSR1 protein.

The *SsGSR1* or *SsGSR1-2* cDNA was subcloned into the EcoRІ/BamHІ sites of the pMAL-c2X expression vector and transformed into the E. coli strain Rosetta(DE3) for the production of recombinant proteins. Positive colonies were selected on 200 μg/mL ampicillin. After induction with 0.1 mM isopropyl-β-d-thiogalactoside (IPTG), the proteins of interest were purified with the Ni-Sepharose FF column.
