# Supplementary material for: Variable Tandem Glycine-Rich Repeats Contribute to Cell Death-Inducing Activity of a Glycosylphosphatidylinositol-Anchored Cell Wall Protein That Is Associated with the Pathogenicity of Sclerotinia sclerotiorum
Source: Microbiol Spectr. 2023 May 4;11(3):e00986-23. doi: 10.1128/spectrum.00986-23 (PMC10269696; doi:10.1128/spectrum.00986-23)
Supplement: Supplemental file 1 — Supplemental material. Download spectrum.00986-23-s0001.pdf, PDF file, 0.6 MB [file spectrum.00986-23-s0001.pdf]

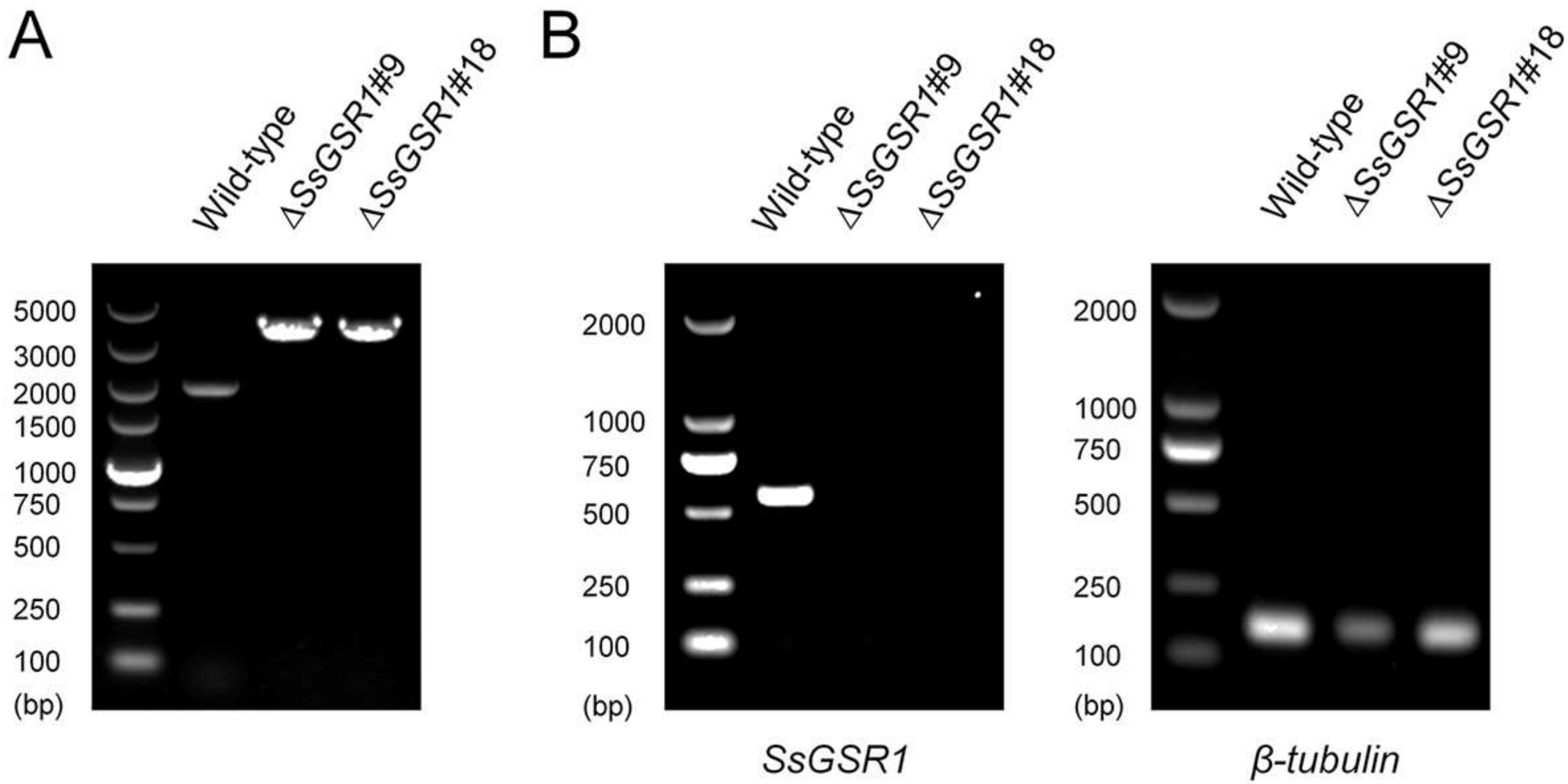

Fig. S1 Disruption of the *SsGSR1* gene in the wild-type strain 1980 using a split-marker strategy. (A) Amplifying the *SsGSR1* band using a primer pair designed beyond the upper and lower homologous fragments from genomic DNA of the wild-type and the two deletion strains, Δ*SsGSR1*#9 and Δ*SsGSR1*#18. (B) RT-PCR analysis of the expressions of *SsGSR1* in the wild-type and two gene-deletion strains. The expression of the *β-tubulin* gene was the control.

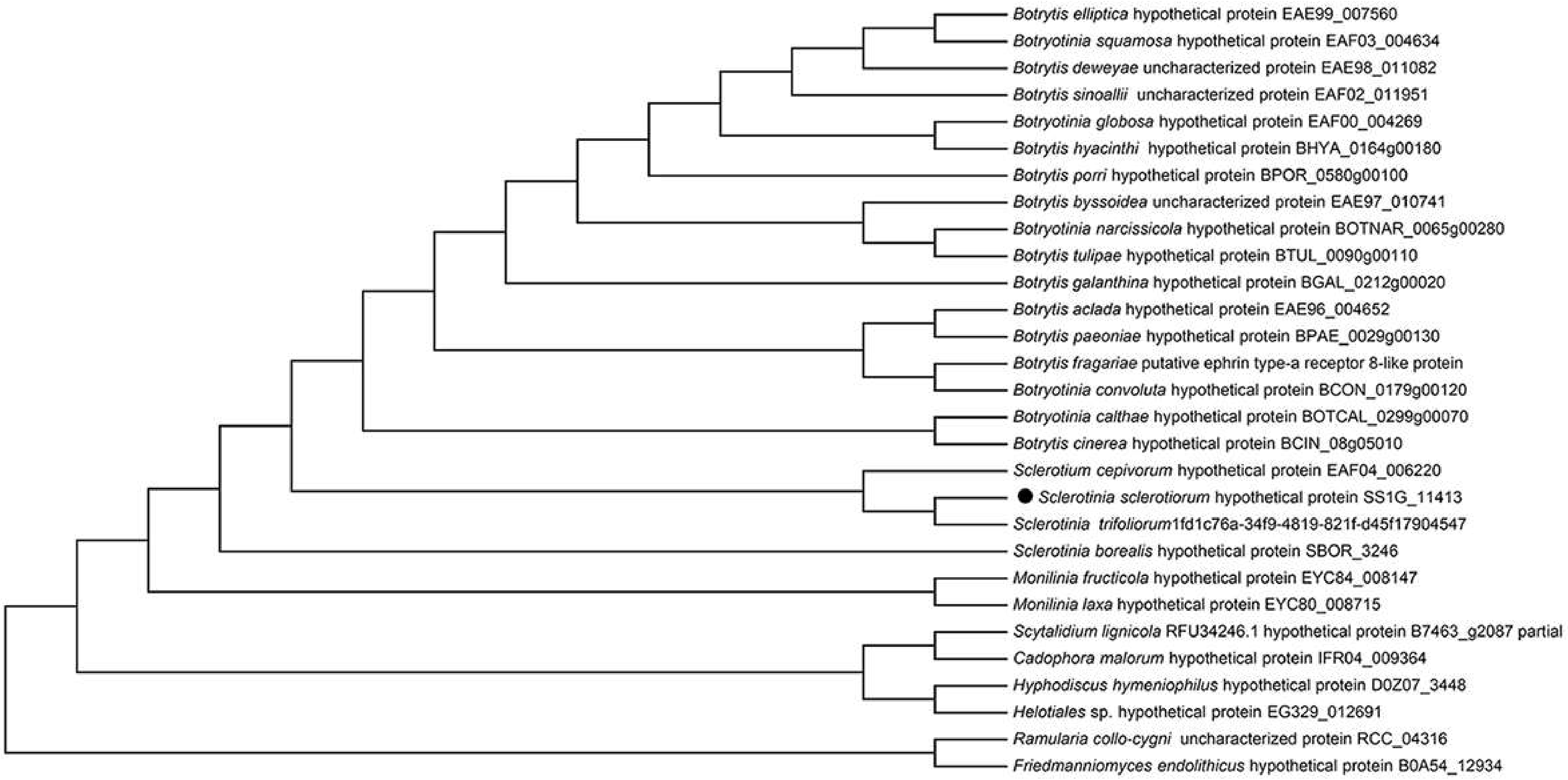

Fig. S2 Phylogenetic analysis of SsGsr1. The phylogenetic tree was constructed using the neighbor-joining method based on the amino acid multiple sequence alignment of SsGsr1 homologous.

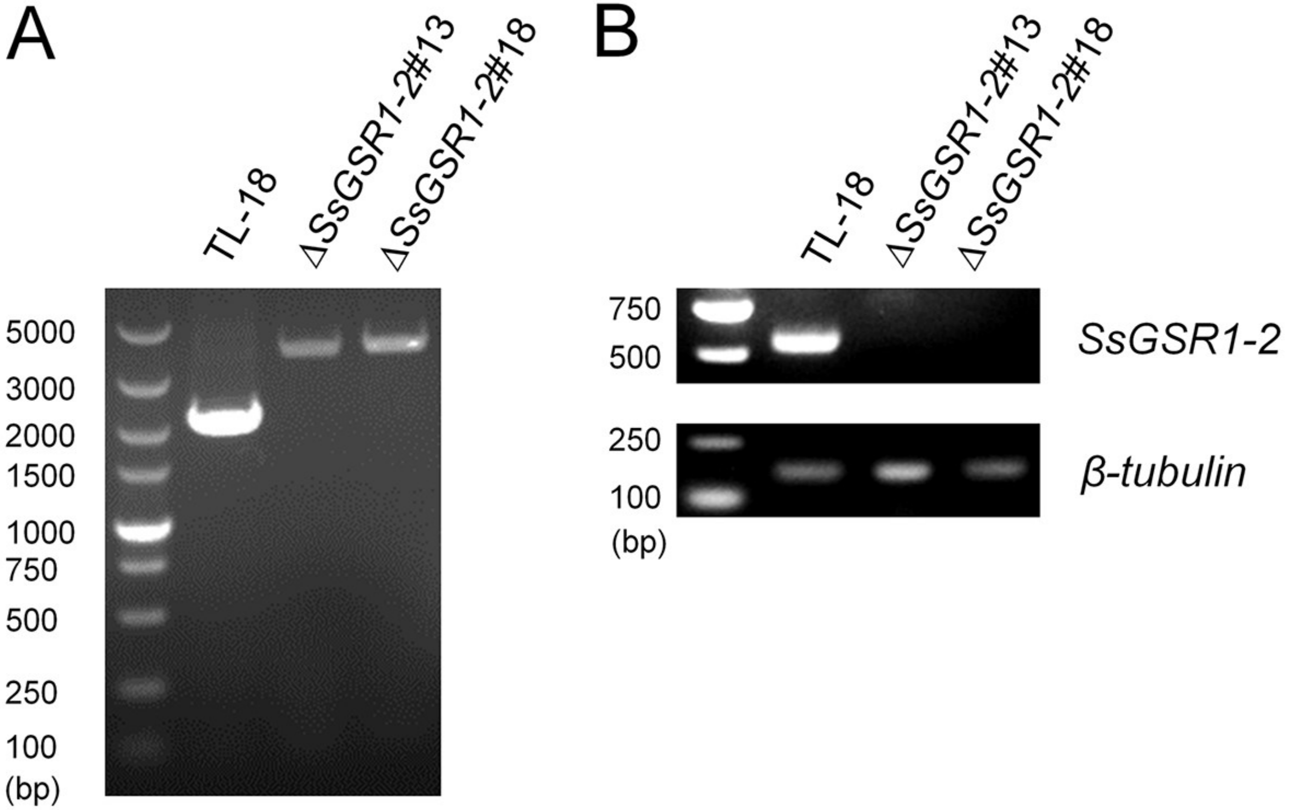

Fig. S3 Disruption of the *SsGSR1-2* gene in the wild-type strain TL-18 using the split-marker strategy. (A) Amplification of *SsGSR1-2* band with the primer pairs designed above (Fig S1A) from genomic DNA of the wild-type strain TL-18 and the gene-deletion strains Δ*SsGSR1-2*#13 and Δ*SsGSR1-2*#18. (B) The expressions of *SsGSR1-2* in TL-18 and two gene-deletion strains. The expression of the *β-tubulin* gene was the control.

Table S1 Amino acid sequences of the proteins for multiple alignments.

Name	Species	Accession No.	Amino acid sequences
SsGsr1	*Sclerotinia sclerotiorum*	XP_001587421.1	MRYQILPAIALLSIGASAQDLGSIVNSITSDVGGALSTATSAVGGAVDTVTSGAGGVFSTVTSGAGGVF<br>STVTSGAGGVFSTVTSGAGGVFSTVTSGAGGAFTTVTSGAGGVFSTVTSGAGGAFSTATSGAGGAFS<br>TATSDAAGAYSSITSAAASKFSSIQSDASSEISSLSSVAATATDSAVKSSISAAMSSISSDVASASSDVRSA<br>TSRASSDFSSATRSAASATSTSSSGAMRTQVPIAGGAILGAALYLL
StGsr1	*Sclerotinia trifoliorum*	CAD6443903.1	MRYQILPAIALLSIGASAQNLGSIVNSITSDVGGALSTATSAIGGAVDTVTSGAGGVFSTVTSGAGGAF<br>STVTSGAGGVFSTVTSGADGAFTTVTSGAGGVLSTVTSGAGGAFSTATSGAAGAFSTATSDAASAYS<br>SITSAAASKFSSIQSGASSEISSLSSVAATATDSAVKSSISAAMSSISSDVASASSGVRSATSRASSDFSSAT<br>KSAASATSTSSSGAMRTQVPIAGGAILGAALYLL
ScGsr1	*Sclerotium cepivorum*	KAF7865243.1	MHYKVLPVIALLSLGASAQDLGSIINSITSDAGGALSTATSAVGGVFSTVTSGAGGVFSTVTSGAGGV<br>FSTVTSGAGGVFSTVTSGAGGAFSTVTSGAGGAFSTVTSGAGGVLSTVTSGAAGAFSTATSDAAGAY<br>SSITSAAASRFSSIQSDAKSEISSLSSVAATATDSAVRSSISAAMSSISSDVASASSAVRSATSKASGDFSS<br>ATKSAASATSSSGAMRTQVPIAGGAILGAALYLL
BbGsr1	*Botrytis byssoidea*	XP_038727874.1	MRVQVLPVIALLSLGVSAQDLGSIVNSITSDVGGAVSTATSAIGGAADTVTSGAAGVFSTVTSGAAGA<br>FSTVTSGAAGAFSTVTSGAGGVVSTITSGAGGVASTVTSGAAGAFSTATSGAAGAFSTATSDAVGAFS<br>TVTSAAASRYSSIKSEASSEIASLSSVAKTATDSAVKSSISAAMSSISSDVASASSEVRSATSRASGALSS<br>ATNSAASATSSSGAMRTQVPMAGGAILGAALYLL
BnGsr1	*Botryotinia narcissicola*	TGO66242.1	MRVQVLPVIALLSIGVSAQDLGSLVNSITSDVGGAVSTATSAIGGAADTVTSGAAGVFSTVTSGAAGA<br>FSTVTSGAGGVVSTVTSGAAGAFSTVTSGAGGVVSTVTSGAAGAFSTATSGAAGAFSTATSDAVGAF<br>STATSAAASRYSSIKSEASSEISSLSSVAQTATDSAVKSSISAAMSSISSDVASASSEVKSATSRASGAVSS<br>ATKSAASATSSSGAMRTQVPMAGGAILGAALYLL
BgaGsr1	*Botrytis galanthina*	THV49076.1	MRVQVLPVIALLSLGVSAQDLGSLVNSITSDVGGAVDTATSAIGGAADTVTSGAAGVFSTVTSGAAG<br>AFSTVTSGAGGVVSTITSGAAGAFSTVTSGAGGVVSTVTSGAAGAFSTATSGAAGAFSTATSDAVGA<br>FSTATSAAASRYSSIKSEASSEISSLSSVAQTATDSAVKSSISAAMSSISSNVASASSEVKSATSRASGAVS<br>SATNSAASATSSSGAMRTQVPMAGGAILGAALYLL

BtGsr1	*Botrytis tulipae*	TGO12320.1	MRVQVLPVIALLSLGVSAQDLGSIVNSITSDVGGAVSTATSAIGGAADTVTSGAAGVFSTVTSGAAGV<br>FSTVTSGAGGVVSTVTSGAAGAFSTVTSGAGGVVSTVTSGAAGAFSTATSDAAGAFSTATSDAVGAF<br>STATSAAASRYSSIKSEASSEISSLSSVAQTATDSAVKSSISAAMSSISSDIVSASSEVKSATSRASGALSS<br>ATKSAASATSSSGAMRTQVPMAGGAILGAALYLL
BglGsr1	*Botryotinia globosa*	KAF7899933.1	MRVQVLPVIALLSIGASAQNLGSLVNAITSDVGGAISTATSAIGGAADTVTSGAAGVFSTVTSGAAGA<br>FSTVTSGAGGVFSTVTSGAAGAFSTATSGAGGVFSTVTSGAAGAFSTATSGAAGAFSTATSDAVGAFS<br>TATSAAASRYSSIKSEASSEISSLSSVAQTATDSAVKSSISAAMSSISSDVASASSEVKSATSSASGALSSA<br>TKSAASATSSSGAMRTQVPMAGGAILGAALYLL
BdGsr1	*Botrytis deweyae*	XP_038804895.1	MRVQVLPVIALLSLGVSAQDLGSIVNSITSDVGGAVSTATSAIGGAADTVTSGAAGVFSTVTSGAAGA<br>FSTVTSGAAGAFSTVTSGAGGVVSTITSGAGGVASTVTSGAAGAFSTATSGAAGAFSTATSDAVGAFS<br>TVTSAAASRYSSIKSEASSEIASLSSVAKTATDSAVKSSISAAMSSISSDVASASSEVRSATSRASGALSS<br>ATNSAASATSSSGAMRTQVPMAGGAILGAALYLL
BeGsr1	*Botrytis elliptica*	KAF7921797.1	MRVQVLPVIALLSLGVSAQDLGSIVNSITSDVGGAVSTATSAIGGAADTVTSGAAGVFSTVTSGAAGA<br>FSTVTSGAAGAFSTVTSGAGGVVSTITSGAGGVASTVTSGAAGAFSTATSGAAGAFSTATSDAVGAFS<br>TVTSAAASRYSSIKSEASSEIASLSSVAQTATDSAVKSSISAAMSSISSDVASASSEVRSATSRASGALSS<br>ATNSAASATSSSGAMRTQVPMAGGAILGAALYLL
BsqGsr1	*Botryotinia squamosa*	KAF7867801.1	MRVQVLPVIALLSLGVSAQDLGSIVNSITSDVGGAVSTATSAIGGAADTVTSGAAGFFSTVTSGAAGA<br>FSTVTSGAGGVVSTITSGAGGVVSTITSGAGGVASTVTSGAAGAFSTATSGAAGAFSTATSDAVGAFS<br>TVTSAAASRYSSIKSEASSEIASLSSVAQTATDSAVKSSISAAMSSISSDVASASSEVRSATSRASGALSS<br>ATNSAASATSSSGAMRTQVPMAGGAILGAALYLL
BcGsr1	*Botrytis cinerea*	XP_001552380.1	MRVQVLPVIALLSLGASAQDLGSLVNSITSDVGGAVDTATSAIGGAVDTVTSGAGGVVSTITSGAGGV<br>FSTVTSGAGGVFSTVTSGAGGVVSTITSGAGGAFSTATSGAAGAFSTATSDAAGAFSTATSAAASRFSS<br>IKSEASSEISSLSSVAATATDSAVKSSISAAMSSISSDVASASSDVKSATSRASSALGSATSSAASATSSSG<br>AMRTQVPMAGGAILGAALYLL
BhGsr1	*Botrytis hyacinthi*	TGO35274.1	MRVQVLPVIALLSLGASAQDLGSLVNSLTSDVGGAINTATSAIGGAADTVTSGAAGVFSTVTSGAAG<br>AFSTVTSGAGGVVSTITSGAGGVFSTVTSGAAGAFSTATSGAAGAFSTATSDAVGAFSTVTSAAASRY

			SSIESEASSEISSLSSVAQTADNAVKSSISAAMSSISSDVASASSEVKSATSRASSALSSATKSAASATSSSGAMRTQVPMAGGAILGAALYLL
BpoGsr1	*Botrytis porri*	TGO83881.1	MRVQVLPVIALLSLGASAQDLGSIVNSITSDIGGAVSTATSAIGGAADTVTSGAAGVFSTVTSGAAGAFSTVTSGAGGVVSTITSGAGGVVSTVTSVAAGAFSTATSGAAGAFSTASSYAVSAFSTVTSAAASRYSSIKSEASSEISSLSSVAQTATDIAVKSSISAAMSSISSDVASASSEIKSATSRASSALSSATGSAASATSKSGAMRTQVPMAGGAILGAALYLL
BsiGsr1	*Botrytis sinoallii*	XP_038752174.1	MRVQVLPVIALLSLGVSAQDLGSIVNSITSDVGGAVSTATSAIGGAADTVTSGAAGVFSTVTSGAAGAFSTVTSGAAGVVSTITSGAGGVASTVTSGAAGAFSTATSGAAGAFSTATSDAVGAFSTVTSAAASRYSSIKSEASSEISSLSSVAQTATDSAVKSSISAAMSSISSDVASASSEVRSATSKASGALSSATKSAASATSSSGAMRTQVPMAGGAILGAALYLL
BaGsr1	*Botrytis aclada*	KAF7955728.1	MRVQVLPVIALLSLGVSAQDLGSIVNSITSDVGGAVDTATSAIGGAVDTVTSGAGGVFSTVTSGAAGAFSTVTSGAGGVISTITSGAGGVASTVTSGAAGAFSTATSGAAGAFSTATSDAVGAFSTATSAAASRYSSIKSEASSEIASLSSVAQTVTDSAVKSSISAAMSSISSDVASASSEVKSATSRASGALSSATNSAASATSSSGAMRTQVPMAGGAILGAALYLL
BpaGsr1	*Botrytis paeoniae*	TGO28285.1	MRVQVLPVIVLLSLGASAQDLGSIVNSITSDIGGAVDTATSAIGGAVDTVTSGAGGVFSTVTSGAAGAFSTVTSGAGGVVSTITSGAGGVASTVTSGAAGAFSTATSGAAGAFSTATSDAVGAFSTVTSAAASRYSSIKSEASSEISSLSSVAQTATDSAVKSSISAAMSSISSDVASASSEVKSATSRASGALSSATNSAASATSSSGAMRTQVPMAGGAILGAALYLL
BfGsr1	*Botrytis fragariae*	XP_037196256.1	MRVQVLPVIALLSLGASAQDLGSIVNSITSDIGGAVDTATSAIGGAVDTVTSGAGGVFSTVTSGAAGAFSTVTSGAGGVVSTITSGAGGVVSTVTSGAAGAFSTATSGAAGAFSTATSDAVGAFSTATSAAASRYSSIKSEASSEISSLSSVAQTATDSAVKSSISAAMSSISSDVASASSEVKSATSKASSALSSATNSAASATSSSGAMRTQVPMAGGAILGAALYLL
BcoGsr1	*Botryotinia convoluta*	TGO50631.1	MRVQVLPVIALLFLGASAQDLGSIVNSITSDVGGAVDTATSAIGGAVDTVTSGAGGVFSTVTSGAAGAFSTVTSGAGGVVSTITSGAGGVVSTVTSGAAGAFSTATSGAAGAFSTATSDAVGAFSTATSAAASRYSSIKSGASSEISSLSSVAQTATDSAVKSSISAAMSSISSDVASASSEVKSATSRASSALSSATDSAASATSSSGAMRTQVPMAGGAILGAVLYLL

BcaGsr1	*Botryotinia calthae*	TEY48012.1	MRVQVLPVIALLSLGASAQDLGSLVNSLTSDIGGAVDTATSAIGGAVDTVTSGAGGVFSTVTSGAGGV<br>FSTVTSGAGGVVSTITSGAGGVVSTITSGAGGAFSTATSGAAGAFSTATSDAAGAFSTVTSAAASKFSS<br>IKSEASSEISSLSSVAATATDSAVKSSISAAMSSISSEVASASSDVKSATSRASSALSSATNSAASATSSSG<br>AMRTQVPMAGGAILGAALYLL
MfGsr1	*Monilinia fructicola*	KAA8567674.1	MGTTVNILIMRYQVLPVVALLSLCASAQDLGDIVNSINSDINGALSTASSALGGAFSSATSDAAGALST<br>ALSNGSGFLSTATSLGAGALSTATSLGAGAFSTATSKAAGEYSTLTSAAASKYSSIQSAASSQISSLSSV<br>AETATDSAIKSSISAAIGSISSDMASASLAISSAASRASSKFNSATSGAATATSTTSNSGAMRTQVPIAGG<br>ALLGAALYLL
MlGsr1	*Monilinia laxa*	KAB8295894.1	MRYQVLPVVALLSLCASAQDLGDIVNSINSDINGALSTASTAFGGAFSSATSNAAGALSTALSNGAGA<br>LSTATSLGDQWLSTATSLGAGALSTATSKAAAEYSTLTSAAASKYSSIQSAANSQISSLNSVAATATDS<br>AIKSSISAAVGSISSDVASASSAISSAASRASSNLNSATSGAAATATSTTSDSGAMKTHVPIAGALLGAA<br>LYLL

Table S2. Primer pairs used in this study.

**Primer name**	**Forward primer (5'-3')**	**Reverse primer (5'-3')**	**Original vector**	**Use**
pGR106-SsGSR1ClaIF/pGR106-SsGSR1HisSalIR	CCCATCGATATGCGTTACCAAATTCTTCCAGCTATT	ACGCGTCGACTCAATGGTGATGGTGATGATGCAACAAGTACAAAGCCGCAC	pGR106	Amplifying the coding region of SsGsr1 for transient expression
pGR106-SsGSR1ΔSPClaIF/pGR106-SsGSR1HisSalIR	CCCATCGATATGCAAGATCTCGGAAGCATCGTCAA	ACGCGTCGACTCAATGGTGATGGTGATGATGCAACAAGTACAAAGCCGCAC	pGR106	Amplifying partial coding region of SsGsr1 (without signal peptide) for transient expression
pGR106-SsGSR1 NClaIF/pGR106-SsGSR1 NHisSalIR	CCCATCGATATGCGTTACCAAATTCTTCCAGT	CGCGTCGACTCAAGCGTAATCTGGAACATCGTATGGGTAATAAGCTCCAGCGGCGTC	pGR106	Amplifying the coding region of SsGsr1$^{1\text{-}146}$ for transient expression
pGR106-SsGSR1 CClaIF/pGR106-SsGSR1 CHisSalIR	CGCATCGATATGCGTTACCAAATTCTTCCAGCTATTGCTCTCCTTTCCATCGGTGCTTCCGCACAAGATCTCGGAAGC TCT TCA ATT ACT TCT GCC GC	CGCGTCGACTCAAGCGTAATCTGGAACATCGTATGGGTACAACAAGTACAAAGCCGCAC	pGR106	Amplifying the coding region of SsGsr1$^{1\text{-}23,\ 147\text{-}254}$ for transient expression
pGR106-SsGSR1 33ClaIF/pGR106-SsGSR1 157HisSalIR	CGCATCGATATGCGTTACCAAATTCTTCCAGCTATTGCTCTCCTTTCCATCGGTGCTTCCGCACAAGATCTCGGAAGCGGCGGCGCGTTGAGCACG	ACGCGTCGACTCAATGGTGATGGTGATGATGGAATTTGGATGCAGCGGCAGA	pGR106	Amplifying the coding region of SsGsr1$^{1\text{-}23,\ 33\text{-}157}$ for transient expression

pGR106-SsGSR1<br>33ClaIF/pGR106-<br>SsGSR1135HisSalIR	CGCATCGATATGCGTTACCAAAT<br>TCTTCCAGCTATTGCTCTCCTTT<br>CCATCGGTGCTTCCGCACAAGA<br>TCTCGGAAGCGGCGGC<br>GCGTTGAGCACG	ACGCGTCGACTCAATGGTGAT<br>GGTGATGATGAAAAGCACCA<br>CCTGCTCCTGA	pGR106	Amplifying the coding region of SsGsr1$^{1-23,3\ 3-135}$ for transient expression
pGR106-SsGSR1<br>33ClaIF/pGR106-<br>SsGSR1113HisSalIR	CGCATCGATATGCGTTACCAAAT<br>TCTTCCAGCTATTGCTCTCCTTT<br>CCATCGGTGCTTCCGCACAAGA<br>TCTCGGAAGCGGCGGC<br>GCGTTGAGCACG	ACGCGTCGACTCAATGGTGAT<br>GGTGATGATGGAAAACTCCAC<br>CTGCTCCCGA	pGR106	Amplifying the coding region of SsGsr1$^{1-23,\ 33-113}$ for transient expression
pGR106-SsGSR1<br>48ClaIF/pGR106-<br>SsGSR1135HisSalIR	CGCATCGATATGCGTTACCAAA<br>TTCTTCCAGCTATTGCTCTCCTT<br>TCCATCGGTGCTTCCGCACAAG<br>ATCTCGGAAGCGATACTGTTAC<br>TTCTGGG	ACGCGTCGACTCAATGGTGAT<br>GGTGATGATGAAAAGCACCAC<br>CTGCTCCTGA	pGR106	Amplifying the coding region of SsGsr1$^{1-23,\ 48-135}$ for transient expression
pGR106-SsGSR1<br>70ClaIF/pGR106-<br>SsGSR1135HisSalIR	CGCATCGATATGCGTTACCAAA<br>TTCTTCCAGCTATTGCTCTCCTT<br>TCCATCGGTGCTTCCGCACAAG<br>ATCTCGGAAGCTCAACTGTTAC<br>GTCTGGC	ACGCGTCGACTCAATGGTGAT<br>GGTGATGATGAAAAGCACCAC<br>CTGCTCCTGA	pGR106	Amplifying the coding region of SsGsr1$^{1-23,\ 70-135}$ for transient expression
pGR106-BcGSR1<br>ClaIF/pGR106-<br>BcGSR1NotIR	CGCATCGATATGCGTGTCCAAG<br>TTCTCCCAG	ATAAGAATGCGGCCGCCTACA<br>ACAAGTACAAAGCCGCACC	pGR106	Amplifying the coding region of BcGsr1 for transient expression
pGR106-MfGSR1<br>ClaIF/pGR106-<br>MfGSR1SalIR	CGCATCGATATGGGTACCACGG<br>TCAATATTCTC	ACGCGTCGACCTACAACAAGT<br>ATAGAGCCGCACC	pGR106	Amplifying the coding region of MfGsr1 for transient expression
pGR106-TL18<br>ClaIF/pGR106-TL18<br>HisSalIR	CGCATCGATATGCGTTACCAAA<br>TTCTTCCAGCT	CGCGTCGACTCAAGCGTAATC<br>TGGAACATCGTATGGGTACAA<br>CAAGTACAAAGCCGCAC	pGR106	Amplifying the coding region of SsGsr1-2 for transient expression

pGR106-EGFPClaIF/pGR106-EGFPHASalR	CGCATCGATATGGTGAGCAAGGGCGAG	CGCGTCGACTCAAGCGTAATCTGGAACATCGTATGGGTACTTGTACAGCTCGTCCATGC	pGR106	Amplifying the coding region of GFP for transient expression
3×HAGSR1HindIII/3×HAGSR1KpnI	CGCAAGCTT ATGCGTTACCAAATTCTTCCAG	CGCGGTACC TTACAACAAGTACAAAGCCGCAC	pSilent-1	Amplifying the coding region of 3×HA-SsGSR1 fusion fragment
pMAL-SsGSR1 EcoRIF/pMAL-SsGSR1BamHIR	GGATCGAGGGAAGGATTTCAGAATTCATGCAAGATCTCGGAAGCATCGTCAATTC	GCCTGCAGGTCGACTCTAGAGGATCCTTACAACAAGTACAAAGCCGCACCCAAAATG	pMAL-c2X	Amplifying the coding region of SsGsr1 for prokaryotic expression
SsGSR1U SacIF/SsGSR1USalIR	CGCGAGCTCCATGATCAACGAGCCACATCG	CGCGTCGACCCACCAGCCCCAGAAGTAAC	pSKH	Amplifying the 5'UTR of *SsGSR1*
SsGSR1DSmaIF/SsGSR1DKpnIR	TGCCCCGGGGCGACTTCAGTTCCGCAACA	CGCGGTACCGTCCTTCGTGCCTATGGTTAACTAG	pSKH	Amplifying the 3'UTR of *SsGSR1*
SsGSR1U/Hy	CATGATCAACGAGCCACATC	AAATTGCCGTCAACCAAGCTC		Amplifying the split-marker fragment for *SsGSR1* deletion
Yg/SsGSR1D	TTTCAGCTTCGATGTAGGAGG	GTCCTTCGTGCCTATGGTTAACTAG		Amplifying the split-marker fragment for *SsGSR1* deletion
SsGSR1KOfp/ SsGSR1KOrp	TGATACTTGAAATTGCTTTGTCCC	TCGGTTTGTCTAAGATTGTTCTGACT		Verification of *SsGSR1* or *SsGSR1-2* deletion strains
RT-SsGSR1F/RT-SsGSR1R	GACGCCGCTGGAGCTTATT	GGCCACATCGCTGCTGATA		Amplifying partial coding region of SsGSR1 for qRT-PCR analysis
RT-tubF/RT-tubR	GTGAGGCTGAGGGCTGTGA	CCTTTGGCGATGGGACG		Amplifying partial coding region of *beta-tubulin* gene (as *S. sclerotiorum* housekeeping gene) for qRT-PCR analysis

NbActin qpcrF/NbActin qpcrR	TGGACACAGGGACTTCATCA	CAAGGGTGAAAGCAAGCAAT		Amplifying partial coding region of actin gene (as *N. benthamiana* housekeeping gene) for qRT-PCR analysis
NbPR1 qpcrF/NbPR1 qpcrR	CCGCCTTCCCTCAACTCAAC	GCACAACCAAGACGTACTGA G		Amplifying partial coding region of PR-1 of *N. benthamiana* for qRT-PCR analysis
NbPR2 qpcrF/NbPR2 qpcrR	AGGTGTTTGCTATGGAATGC	TCTGTACCCACCATCTTGC		Amplifying partial coding region of PR-2 of *N. benthamiana* for qRT-PCR analysis
